# The Performance of Gene Expression Signature-Guided Drug–Disease Association in Different Categories of Drugs and Diseases

**DOI:** 10.3390/molecules25122776

**Published:** 2020-06-16

**Authors:** Xiguang Qi, Mingzhe Shen, Peihao Fan, Xiaojiang Guo, Tianqi Wang, Ning Feng, Manling Zhang, Robert A. Sweet, Levent Kirisci, Lirong Wang

**Affiliations:** 1 Department of Pharmaceutical Sciences, Computational Chemical Genomics Screening Center, University of Pittsburgh School of Pharmacy, 3501 Terrace St Pittsburgh, PA 15261, USA; Xiq24@pitt.edu (X.Q.); MIS216@pitt.edu (M.S.); pef14@pitt.edu (P.F.); xig53@pitt.edu (X.G.); 2 Department of Biological Sciences, University of Pittsburgh School of Arts & Sciences, Pittsburgh, PA 15260, USA; Lu_Wang@pitt.edu; 3 Division of Cardiology, Vascular Medicine Institute, University of Pittsburgh School of Medicine, Pittsburgh, PA 15213, USA; FENGN@pitt.edu (N.F.); zhangm5@upmc.edu (M.Z.); 4 Department of Neurology, University of Pittsburgh School of Medicine, Pittsburgh, PA 15213, USA; 5 Department of Psychiatry, University of Pittsburgh School of Medicine, Pittsburgh, PA 15213, USA

**Keywords:** gene expression signature, drug repositioning approaches, RNA expression regulation

## Abstract

A gene expression signature (GES) is a group of genes that shows a unique expression profile as a result of perturbations by drugs, genetic modification or diseases on the transcriptional machinery. The comparisons between GES profiles have been used to investigate the relationships between drugs, their targets and diseases with quite a few successful cases reported. Especially in the study of GES-guided drugs–disease associations, researchers believe that if a GES induced by a drug is opposite to a GES induced by a disease, the drug may have potential as a treatment of that disease. In this study, we data-mined the crowd extracted expression of differential signatures (CREEDS) database to evaluate the similarity between GES profiles from drugs and their indicated diseases. Our study aims to explore the application domains of GES-guided drug–disease associations through the analysis of the similarity of GES profiles on known pairs of drug–disease associations, thereby identifying subgroups of drugs/diseases that are suitable for GES-guided drug repositioning approaches. Our results supported our hypothesis that the GES-guided drug–disease association method is better suited for some subgroups or pathways such as drugs and diseases associated with the immune system, diseases of the nervous system, non-chemotherapy drugs or the mTOR signaling pathway.

## 1. Introduction

A gene expression signature (GES) is a set of comprehensive gene expression profiles that can reveal the difference between stimulated and normal cell states [1]. Current applications of GES analysis are fruitful in cancer-related areas for disease genotype classification and outcome predictions. For example, Ramaswamy, S. et al. created a GES database for diagnosing and categorizing the tumour type with an accuracy rate of 78% [2]. Wright, G. et al. developed a Bayesian rule-based algorithm to classify diffuse large B cell lymphoma into two subgroups which have a significant difference in the five-year survival rate [3]. Although the GES method is more commonly used in diagnosing cancer [2,3,4,5] and predicting the outcome of certain medical interventions [6,7], some successful cases of application on drug development have also reported [8,9,10].

Generally, there are two major strategies for applying GES analysis on drug development: drug–drug-based and drug–disease-based. The drug–drug-based method determines the mechanistic actions of drugs by comparing the similarity between the GES induced by a drug of interest to those of drugs with known mechanisms. If two different drugs have similar GES profiles, then they are considered to have “functional similarity”, meaning they work in a similar manner. In contrast, the drug–disease-based method compares the similarity between the GES of a drug to that of a disease in order to determine its potential as a new therapeutic agent. If the GES profile of the drug is opposite to opposite to the expression pattern of the disease, then the drug is considered to have a therapeutic effect for the disease. However, if they have similar patterns, then this drug may exacerbate the disease. Studies aimed at drug repurposing or repositioning based on GES analysis usually use one or both of these strategies [8,9,10]. In addition, there are studies trying to combine the GES method with other methods, such as machine learning, to increase the accuracy of compound indication prediction [11]. However, as those kinds of GES-guided drug repurposing studies usually just reported the successful predicted cases, therefore, the true accuracy of these methods needs to be assessed.

Due to the different and complex mechanisms of disease processes, the idea of an “inverse pattern of a GES between drugs and diseases for therapeutic effect” may not hold, or at least may not be suitable for all categories of drugs and diseases. In other words, a GES may be useful for certain diseases, but not for others. To our knowledge, the application domains of GES-guided drug–disease associations have not been reported. Herein, we conducted a study to validate the power of the GES-guided drug repositioning method and to further explore which specific subgroups of drug–disease pairs are more suitable for this method. Moreover, the most significant subgroup was selected as a case report of detailingwhich genes and/or pathways were more sensitive to the GES-guided drug repositioning method.

## 2. Results

### 2.1. GES Profiles Enrollment and Drug–Disease Pairs

**Figure 1:**
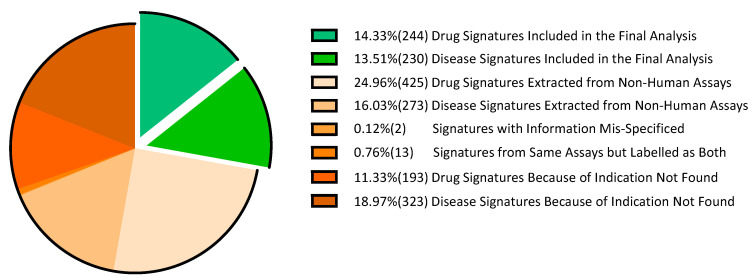
The proportion of data sourced from the crowd extracted expression of differential signatures (CREEDS) database. Numbers of gene signatures are shown in parentheses. “Drug and Disease Signatures Included in the Final Analysis”: The proportion of drug or disease gene signatures enrolled in the final analysis. “Drug and Disease Signatures Extracted from Non-Human Assays”: The proportion of drug or disease gene signatures extracted from non-human assays. “Signatures with Information Mis-Specified”: The proportion of gene signatures with information errors. “Signatures from Same Assays but Labelled as Both”: The proportion of gene signatures excluded because of both drug and disease sourcing from the same assay. “Drug and Disease Signatures Because of Indication Not Found”: The proportion of gene signatures excluded because no FDA-labelled indication of a relationship was found for the drug or disease (including drugs not approved by FDA).

After removing signatures from non-human assays and signatures of non-FDA (the U.S. Food and Drug Administration)-approved drugs, we found that GSE10432, GSE7036, GSE6264, GSE38713, GSE31773, GSE11393, GSE8157, GSE13887 and GSE11223 were signatures of both drugs and diseases from the same assays. We kept their disease labels except the CREEDS (crowd extracted expression of differential signatures [12]) ID of dz:297 because this case had information mis-specified (wrong disease information with its original experiment). Two GES profiles from mouse (drug:3288 and dz:724) were mis-specified as human and were also excluded from analysis. The relationship between these Gene Expression Omnibus (GEO) series (GSE) and CREEDS IDs is shown in Table 1**.** The proportion of data that meets the inclusion criteria is shown in Figure 1.

When the inclusion criteria were applied, and the signatures with no indication relationship were excluded, 230 manual disease signatures and 244 manual drug perturbation signatures from 71 unique diseases and 56 unique drugs, respectively, were enrolled in the final analysis. The average signed Jaccard indexes [12] (SJI) of 3976 unique drug–disease pairs were calculated. Among them, there were 167 pairs with a drug–disease indication from the drug labels. The remaining 3809 unique drug–disease pairs were used as the control group.

### 2.2. Subgroups Distribution

**Figure 2:**
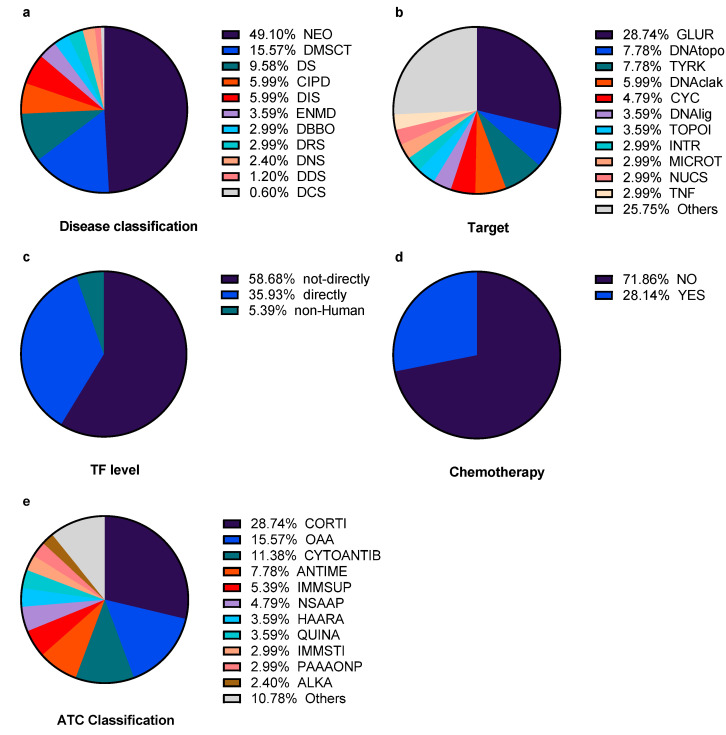
The subgroups proportion of unique 167 indicated drug–disease pairs of different categories. (**a**) Disease classification. NEO: neoplasms, DMSCT: diseases of the musculoskeletal system or connective tissue, DS: diseases of the skin, CIPD: certain infectious or parasitic diseases, DIS: diseases of the immune system, ENMD: endocrine, nutritional or metabolic diseases, DBBO: diseases of the blood or blood-forming organs, DRS: diseases of the respiratory system, DNS: diseases of the nervous system, DDS: diseases of the digestive system, DCS: diseases of the circulatory system. (**b**) Drug target. GLUR: glucocorticoid receptor, DNAtopo: DNA/topoisomerase-human, TYRK: tyrosine kinase, DNAclak: DNA cross-linking/alkylation, CYC: cyclooxygenase, DNAlig: DNA/ligase, TOPOI: topoisomerase-non-human, INTR: interferon receptor, MICROT: microtubules, NUCS: nucleotide synthesis, TNF: tumor necrosis factor. (**c**) TF (transcription factor) level. “Directly”: drugs with TFs as its main therapeutic targets. “Not-directly” indicates drugs with main therapeutic targets which are human DNA structures or human proteins but not TFs. “Non-Human” represent drugs interacting with protein or structures of non-human (for example, from virus or bacterial) as main therapeutic targets. (**d**) Chemotherapy. “YES” or “NO” indicates the drug is a chemotherapy drug or not. (**e**) ATC classification. CORTI: corticosteroids for systemic use, plain, OAA: other antineoplastic agents, CYTOANTIB: cytotoxic antibiotics and related substances, ANTIME: antimetabolites, IMMSUP: immunosuppressants, NSAAP: anti-inflammatory and antirheumatic products, non-steroids, HAARA: hormone antagonists and related agents, QUINA: quinolone antibacterial, IMMSTI: immunostimulants, PAAAONP: plant alkaloids and other natural products, ALKA: alkylating agents.

Among the 56 unique drugs analysed, 32 unique protein targets with 22 categories of Anatomical Therapeutic Chemical (ATC) classification were assigned. Thirteen drugs are classified as chemotherapy drugs, and 44 drugs are not (Methotrexate is both a chemotherapy and a non-chemotherapy drug due to its different main therapeutic targets when against different diseases). For transcription factor (TF) level, 12 drugs are labelled as “directly”, 39 drugs are labelled as “not-directly” and 5 drugs were labelled as “non-Human” (see section *4.4. subgroup classification* for the detailed meanings of labels). Further, 71 diseases are divided into 11 ICD-11 (International Classification of Diseases 11th Revision) categories. In total, 70 subgroups belonging to five categories were assigned (Figure 2, detailed information in Table S1 and Table S2).

### 2.3. Overall Score of GES Similarity of Drug-Indicted Disease Pairs Against Random Drug–Disease Pairs

**Figure 3:**
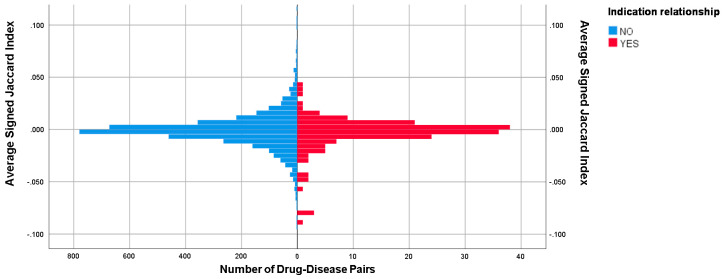
The distribution of signed Jaccard index in the indication group and the control group.

We observed significantly lower SJI similarity scores of drug–disease indication pairs than those of random drug–disease pairs (p-value of two-side t-test [13] equals to 0.02324). The average similarity score of indicated pairs is −0.00386 with a standard deviation of 0.01794 and that of random control pairs is −0.00072 with a standard deviation of 0.01750, indicating that the GES method can reflect the therapeutic effects of the drugs (The distributions of SJI in both the indication group and the control group are shown in Figure 3). 

### 2.4. Subgroup Scores of GES Similarity of Drug-Indicated Disease Pairs Against Random Drug–Disease Pairs

**Figure 4:**
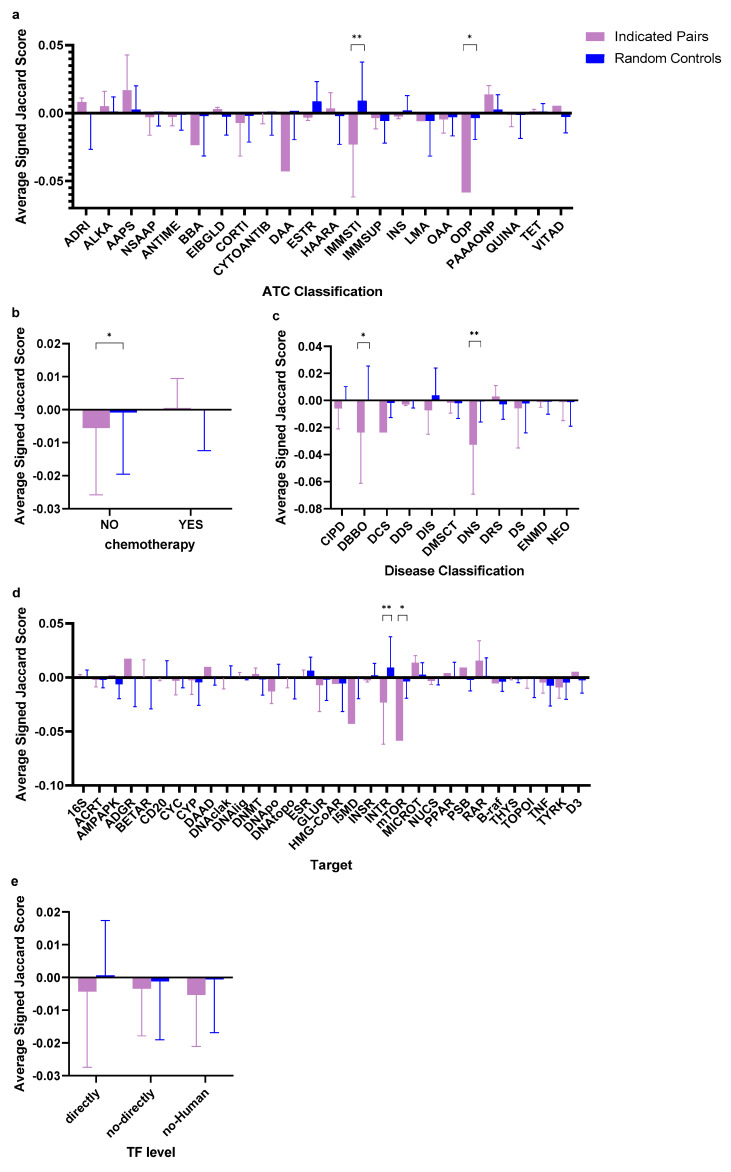
The average signed Jaccard index score of unique indicated drug–disease pairs split by different categories of subgroups. ** indicates FDR Q < 0.01, * indicates FDR Q < 0.05. (**a**) ATC classification. ADRI: adrenergics, inhalants, AAPS: anti-acne preparations for systemic use, EIBGLD: blood glucose-lowering drugs, excluding insulins, DAA: direct acting antivirals, ESTR: estrogens, INS: insulins and analogues, LMA: lipid modifying agents, plain, ODP: other dermatological preparations, TET: tetracyclines, VITAD: vitamins A and D, including combinations of the two. CORTI, OAA, CYTOANTIB, ANTIME, IMMSUP, NSAAP, HAARA, QUINA, IMMSTI, PAAAONP, ALKA, see Figure 1 legend. (**b**) Chemotherapy. “YES” or “NO” indicates the drug is a chemotherapy drug or not. (**c**) Disease classification. See Figure 1 for abbreviations. (**d**) Target. 16S: 16S ribosomal RNA, ACRT: aminoimidazole caboxamide ribonucleotide transformylase, AMPAPK: AMP-activated protein kinase, ADGR: androgen receptor, BETAR: beta adrenergic receptor, CD20: CD20 antigen, CYP: cytochromes P450, DAAD: delta-aminolevulinic acid dehydratase, DNMT: DNA/methyltransferase, DNApo: DNA/polymerase, ESR: estrogen receptor, HMG-CoAR: HMG-CoA reductase, I5MD: inosine-5’-monophosphate dehydrogenase, INSR: insulin receptor, mTOR: kinase mTOR, PPAR: peroxisome proliferator-activated receptors, PSB: proteasome subunit beta, RAR: retinoic acid receptor, B-raf: serine/threonine-protein kinase B-raf, THYS: thymidylate synthase, D3: vitamin D3 receptor; GLUR, DNAtopo, TYRK, DNAclak, CYC, DNAlig, TOPOI, INTR, MICROT, NUCS, TNF see Figure 1 legend. (**e**) TF (transcription factor) level. “Directly”: drugs with TFs as their main therapeutic targets. “Not-directly” indicates drugs with main therapeutic targets which are human DNA structures or human proteins but not TFs. “Non-Human” represents drugs interacting with non-human proteins or structures (for example, from viruses or bacteria) as main therapeutic targets.

We compared drugs from five different categories of subgroups: (1) disease classifications; (2) drug target; (3) TF level; (4) chemotherapy; and (5) ATC classification. The results are shown in Figure 4, detail information is listed in Table S3 and Table S4. Subgroups with important or significant (q-value according to false discover rate (FDR) lower than 0.05) results according to least squares mean partitions F tests of a generalized linear model (GLM) [14] are listed in Table 2. 

### 2.5. Gene and Pathway Analysis on an Example Drug–Disease GES Pair

Interferon receptor (with the same drug–disease pair content as the immunostimulants subgroup), the subgroup with the lowest q-value, was chosen as a case report for the pathway analysis. The top 5% (93/1898) genes with a relatively reversed expression probability according to the relatively expression probability of a gene’s (***G^I-R%^***, an indicator of the relative possibility difference of gene expression between the indicated group and the random control group, see below 4.5) scores are shown in Table 3. The top 10 significant biological pathways identified by the ingenuity pathway analysis are shown in Table 4.

These 10 pathways are reported to be involved with interferon regulation [15,16,17,18,19,20,21,22,23,24,25,26,27]. within inflammatory and immune responses (see Table 5).

## 3. Discussion

It is well-recognized that genes with similar gene expression patterns have a similar function [34]. From the overall score, we can see that FDA-approved drugs listed in the CREEDS database and their indicated diseases generally have inverse GES patterns compared with the random controls. However, the absolute difference between the indicated group and random control group is not very obvious. For example, in a recent study [35], a significant relationship was found between drug–disease GES similarity and drug therapeutic effect using Cmap [36], with a relatively low overall area under curve (AUC) of 0.57, indicating a real, albeit weak, inverse relationship. The treatment effectors of the drugs identified in this study likely work via the interaction of the genes’ protein products, with only a moderate correlation between gene expression and levels of the corresponding protein(s) [37]. Thus, an association study between drugs/diseases and gene expression/pharmaceutical effect is necessary. Also, other mechanisms, for instance, microRNA-based therapeutics, might directly orchestrate the activation/deactivation of the gene expression. However, due to the limitation of available sources, we were unable to investigate other mechanisms of action. Besides, the drugs’ TF-levels were not a significant factor that reflect the indication relationship (although drugs directly interacting with TF perform slightly better with q-value of 0.22309 vs. q-value of 0.99509). In our analyses, some subgroups of drugs–diseases pairs with indication associations have positive similarity scores (which means that the drug may exacerbate the disease according to the assumption of gene expression signature similarity) or a score higher than random drug–disease pairs, but these findings were not statistically significant. On the other hand, 7 of 70 subgroups had a significantly lower similarity score when a drug–disease association is indicated. 

This study may provide some hints to other future studies utilizing the GES method strategies of comparing drug–disease GES similarity for drug repositioning. That is, certain types of drugs may have a stronger ability to reverse the GES of the diseases they treat, and the disease type may also influence this ability. As such, in specific kinds of subgroups, the drug–disease pairs with higher similarities of reversed GES patterns may have greater therapeutic relationships, which means that focusing on certain kinds of diseases or drugs can increase the true positive rate of the GES-guided drug repositioning method For example, over half (4/7) of the significant subgroups (immunostimulants, interferon receptor, other dermatological preparations, and diseases of the blood or blood-forming organs) are related to diseases associated with the immune system (the disease includes in “other dermatological preparations” atopic dermatitis). This indicates that a drug with drug–disease pairs associated with the immune system tends to have lower similarity scores when compared with the diseases it indicated than random diseases. This means in a GES-guided drug repositioning analysis, an immune-associated drug is more likely to have a potential therapeutic effect on diseases that have a higher inverse similarity with it.

Chemotherapy drugs may not be as good as non-chemotherapy drugs for the GES-guided drug repositioning method (q-values: 0.99509 vs. 0.03937). Unarguably, the high diversity of chemotherapy responses to heterogenetic tumor tissues or even histologically similar tumors has been a challenging problem for a long time [37,38]. The failure of controlling the process of programmed cell death in tissue, one of the major causes of tumors, can be rectified or even overturned by activating/deactivating different pathways under various conditions [39]. This may be the reason that chemotherapy drugs are not good for the GES-guided repositioning approach. On the other hand, non-chemotherapy drugs show a significant result as they interact with cancer cells through more specific mechanisms, such as hormone regulation or mono-target therapy.

For the biological pathway analysis of the interferon receptor subgroup, we found that the genes involved in pathways directly regulated by drugs have the lowest GI-R% scores. It is reasonable that GES-guided drug repositioning methods are more sensitive to drugs directly targeting pathways related to diseases. Furthermore, the significance of mTOR signaling is in accordance with the result in which the subgroup kinase mTOR had a significant indicated-random drug–disease pairs’ SJI difference. This result confirmed the high sensitivity of the GES-guided drug repositioning method to this pathway on the other side.

There are some limitations in this study. First, the tissues used for testing the drug effects may not match with the body parts/organs affected by the diseases.. Second, some bias may be caused by the limited number of the CREEDS bio-assay collection which may not have the ability to fully present the pattern of all kinds of drugs and diseases. Additionally, it is important to differentiate the types of “treatment effect”. Some drugs may cure a particular disease while others may just provide symptomatic relief thereby resulting in different patterns of GES for the same disease. Also, some indicated subgroups (“kinase mTOR”, and “other dermatological preparations”) have too few unique drug–disease pairs (n = 1), which may weaken the analyses’ power.

In this study, we systematically analyzed the similarity of gene expression profiles from known drug–disease associations, and we found that indicated pairs have a greater inverse similarity score. We found seven subgroups in which their drugs or diseases may have a greater reversed GES pattern when there is a clear therapeutic effect. These findings suggest that a GES-guided drug repositioning method should be used based on the drug or disease type differences. For example, drugs or diseases associated with the immune system, diseases of the nervous system or non-chemotherapy drugs may be a better choice for drug repositioning. Moreover, our biological pathway enrichment analysis showed that some pathways may be more sensitive to this method, such as the mTOR signaling pathway.

## 4. Materials and Methods

### 4.1. Gene Signature Data Collection and Filtering

In this study, all gene signature information was collected from a well-calibrated GES repository, the crowd extracted expression of differential signatures (CREEDS) [12] database. The CREEDS database is maintained by the Ma’ayan Lab of Icahn School of Medicine at Mount Sinai. CREEDS utilized GEO2Enrichr [40] to extract GES profiles from the National Center for Biotechnology Information (NCBI) Gene Expression Omnibus (GEO) and applied a characteristic direction (CD) model [41] to identify differentially expressed genes. This database V1.0 includes 10,797 single-gene perturbations, 2258 disease signatures and 5516 drug perturbation gene signatures. Among these signatures, 2176 manual single-gene perturbations, 828 manual disease signatures and 875 manual drug perturbation signatures were considered to be more accurate compared with the automatically generated GES by the machine learning method. The CREEDS database allows users to compare the similarity between the user-specified GES and the GESs processed and stored in the CREEDS.

We first selected the CREEDS manual GES profiles if the assays were from human tissues and/or human cell linesand if the drugs had FDA approval. 

**Figure 5:**
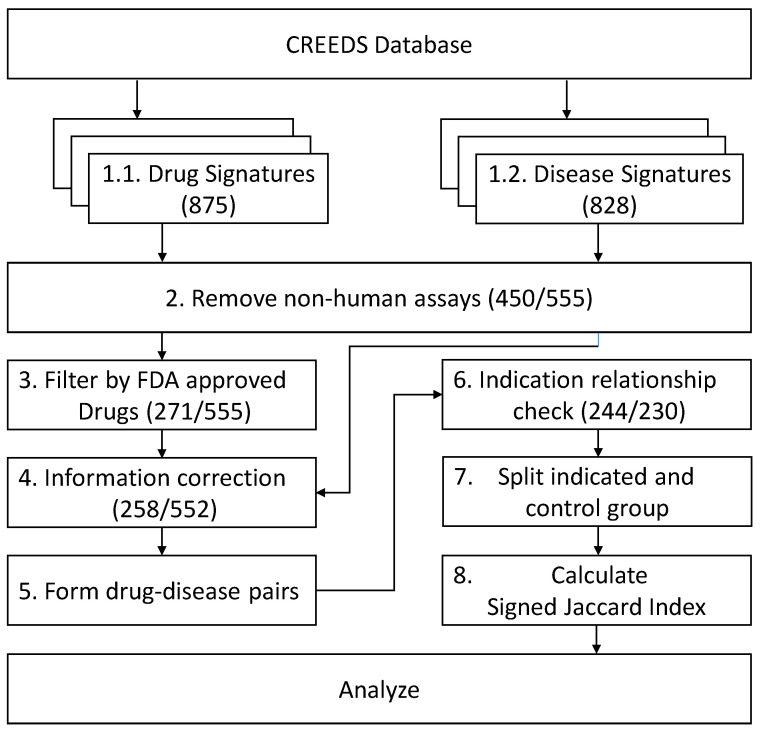
The flow chart of drug and disease gene signature data inclusion process. Numbers of gene signatures left in each step are shown in parentheses: (Number of drug signatures/Number of disease signatures) 1.1. and 1.2. All manual gene signatures retrieved from the CREEDS database. 2. Remove all signatures with assays not labelled as human. 3. Remove all drug signatures not from FDA-approved drugs. 4. Remove signatures with information errors or signatures labelled as both for a drug treatment and for a disease. 5. Remaining drug signatures were paired with each disease signature. 6. Remove signatures with no FDA-labelled indication relationships of drug or disease. 7. Indicated group and control group were divided according to the indication relationship from the FDA drug label. 8. Calculate the signed Jaccard index for each remaining drug–disease pair.

Each GES profile includes a list of up- and down-regulated genes. The SJI [12] (see below), a measurement for the similarity between two GES profiles from the paired drug–disease, was calculated. When a drug or a disease had multiple GES profiles, we calculated the SJIs of all the possible combinations, and an overall score for each unique drug–disease pair was calculated from the average of all scores from pairs sharing the same drug–disease combination. All the disease signatures and drug perturbation signatures were requested through the application program interface (API) provided by CREEDS. GES profiles were removed if they labelled for both a drug treatment and for a disease, because this may cause biased similarity. Under the criteria that (a) the GES profiles must come from assays of human cells/tissues, and (b) drugs must be approved by FDA, the remaining signatures were paired within drugs and diseases according to the indication associations. Signatures without any indicated drug–disease relationship were also excluded from further analysis. For example, cocaine was removed because its indication, local anesthesia, was not in the data of disease signatures and could not be paired. The overall data process is shown in Figure 5.

### 4.2. Similarity Calculation

In our analysis, SJI, which is based on the Jaccard similarity coefficient [42], was used to compute the similarity between GES profiles from a drug and a disease. The Jaccard similarity coefficient is a statistic used to gauge the similarity between different sample sets. It is defined as the size of the intersection divided by the size of the union of two sample sets. It is calculated as follows:

$$Jaccard\;Similarity\;Coefficient\left(G_{1},G_{2}\right)=\frac{\mathrm{SAME}}{\mathrm{ALL}}$$

where G_1_ and G_2_ stand for two lists of differential expressed gene sets, “SAME” represents the number of same genes between two given gene sets, and “ALL” stands for all the unique genes that appeared in the two gene sets. 

SJI, which combines the Jaccard similarity coefficient with the gene regulation direction is calculated as follows:

$$Signed\;Jaccard\;index\left(G_{1},G_{2}\right)=\frac{J\left(G_{1}^{up},G_{2}^{up}\right)+J\left(G_{1}^{down},G_{2}^{down}\right)-J\left(G_{1}^{up},G_{2}^{down}\right)-J\left(G_{1}^{down},G_{2}^{up}\right)}{2}$$

where J means Jaccard similarity coefficient, and G^up^ and G^down^ are up- or down-regulated genes in the given gene set G, respectively. The index ranges from +1 to −1, where +1 and −1 indicate a same pattern and inverse pattern of two gene sets, respectively. Zero indicates that the two sets have no associations, or the same part is cancelled out by the inverse part. The reason to use an unranked score calculation method (SJI) is to keep in accordance with the same scoring method used in the CREEDS database. The CREEDS API (application programming interface) offers the function to calculate the SJI automatically. However, we found the API could not calculate the SJI correctly when two GES profiles are highly overlapped., therefore, all the SJIs in this study were re-calculated.

### 4.3. Drug-Related Information Collection

In our analysis, the source of drug-related information is listed as follows:

1. Drug target information was collected from DrugBank [43,44] Release Version 5.1.4 [45] (https://www.drugbank.ca/releases/latest#external-links). Only the targets with the main therapeutic effect in the mechanism of action section were included;

2. The human TF list was collected from the paper published by Samuel A. Lambert et al. [46]; 

3. ATC classifications on level 3 were collected from the WHO official website (https://www.whocc.no/atc_ddd_index/);

4. The drug indication was from section “indications and usage” of FDA label on FDA website (https://labels.fda.gov/); 

5. (Drug-indicated) Disease classification was assigned to each disease based on the International Classification of Diseases 11th Revision (ICD-11), level 1.

### 4.4. Subgroup Classification

In our analysis, we assessed the following factors that might influence the power of the GES-guided drug repositioning method:

1. Disease classifications: A subgroup was assigned to a disease in a drug–disease pair according to the ICD-11-level 1 code of the disease;
2. Drug target subfamilies: Subgroups were divided by the main therapeutic target of each drug. To avoid group splits being too small, some same subfamilies of targets are grouped as one, such as “Beta-1 adrenergic receptor”, “Beta-2 adrenergic receptor” and “Beta-3 adrenergic receptor” are grouped in the same subgroup “Beta adrenergic receptors”;
3. The relationship between the drug’s main therapeutic targets and human transcription factors: A TF level was assigned according to the relationship between the drugs’ main therapeutic targets and human TF. Drugs with main therapeutic targets that can directly interact with at least one TF were labelled as “directly”. Drugs with main therapeutic targets which are human DNA structures or human proteins but not TFs were labelled as “not-directly”. Drugs interacting with non-human proteins or structures (for example, from viruses or bacteria) as main therapeutic targets were labelled as “non-Human”;
4. The drug is a chemotherapy drug or not: Drugs with main therapeutic targets as “DNA cross-linking/alkylation”, “DNA/ligase”, “DNA/methyltransferase”, “DNA/polymerase”, “DNA/topoisomerase-human”, “micro-tubules”, “nucleotide synthesis” or “Thymidylate synthase” were defined as chemotherapy drugs
5. The drug’s ATC classification: Subgroups were divided according to the Anatomical Therapeutic Chemical classification system, level 3. Drugs with multiple classifications caused by different administration routes were unified to systematic use.

### 4.5. Statistical Analysis and Pathway Analysis

The random control group was generated by calculating the average SJI of all possible drug–disease pairs without indicated associations to imitate a GES-guided drug repositioning screening. A t-test [13] was applied to quantify the mean differences of the SJI between drug-indicated disease pairs and random controls.

For subgroup analysis, GLM [14] least squares mean partitions F tests function was applied to estimate the mean difference between the indicated and control group since the data was unbalanced with multiple factors. A significant result of a certain subgroup indicated that the average SJI of this subgroup was significant between two indication levels (Yes/No). False discovery rate (FDR) q-value of the Benjamini–Hochberg procedure [47] was controlled to 0.05 to avoid an inflated experiment-wise type I error rate caused by multiple comparisons among all subgroups.

Data processing and statistical analysis (student t-tests, GLM, FDR calculation) were conducted using R studio 3.6.1 [48] and SAS software version 9.4. Copyright © 2019 SAS Institute Inc. Cary, NC, USA.

Differentially reversed expression genes (top 5% negative score according to the relatively reverse percentage) from the most significant subgroup will be chosen as examples to conduct biological pathway enrichment analysis.

The relatively reverse percentage is calculated as

$$Relatively\;expression\;probability\;of\;a\;gene\left(G^{I-R\%}\right)=D^{I}\%-D^{R}\%$$

where ***D^I^%*** **and *D^R^%*** stand for the percentage of the gene which is differentially expressed in all assays of indicated/random drug–diseases pairs. It is calculated as

$$D\%=\frac{NS-NR}{Total\;assays\;pairs}$$

where **NS** and **NR** represent the number of times a gene showed a same or reverse regulation direction between assays of drugs and diseases among all drug–disease assays pairs.

The ***G^I-R%^*** ranges from 100% to -100%. A higher positive score indicates that this gene is more likely to be expressed in the same direction in indicated drug–disease assays compared with random drug–disease assays. Likewise, a lower negative score indicates that this gene has a higher probability to express reversely between indicated drug–disease assays compared with random drug–disease assays.

Biological pathway enrichment analysis was conducted by ingenuity pathway analysis (IPA, QIAGEN Inc., https://www.qiagenbioinformatics.com/products/ingenuitypathway-analysis).

## Figures and Tables

**Table 1:** The Gene Expression Omnibus (GEO) series with crowd extracted expression of differential signatures (CREEDS) IDs excluded.

GEO Series	CREEDS IDs	Excluded CREEDS IDs
GSE10432	drug:2772, dz:297	dz:297
GSE7036	drug:3292, dz:181	drug:3292
GSE6264	drug:3064, dz:582	drug:3064
GSE38713	drug:3289, drug:3194, drug:3195, dz:810	drug:3289, drug:3194, drug:3195
GSE31773	drug:2485, dz:712, dz:713, dz:714, dz:715	drug:2485
GSE11393	drug:3401, drug:3196, dz:773, dz:267	drug:3401, drug:3196
GSE8157	drug:2796, dz:880	drug:2796
GSE13887	drug:3181, dz:450	drug:3181,
GSE11223	drug:3294, drug:3287, dz:590, dz:591, dz:593, dz:589, dz:588, dz:587, dz:586, dz:585	drug:3294, drug:3287
GSE7762	drug:3288	drug:3288
GSE3248	dz:724	dz:724

**Table 2:** Subgroups of generalized linear model (GLM) least squares mean partitions F tests results.

Classification Category	Subgroups	Average SJI of Indicated Pairs ± SD	N	Average SJI of Control Pairs ± SD	N	Q value
Disease classification	Diseases of the blood or blood-forming organs	−0.02368 ± 0.03746	6	0.00075 ± 0.02470	138	0.01322
	Diseases of the nervous system	−0.03264 ± 0.03648	4	−0.00054 ± 0.01528	92	0.00704
Drug target classification	Interferon receptor	−0.02314 ± 0.03866	5	0.00916 ± 0.02849	115	0.00110
	Kinase mTOR	−0.05846 ± ----------	1	0.00353 ± 0.01580	23	0.01755
Chemotherapy classification	Chemotherapy drugs	0.00048 ± 0.00894	47	−0.00022 ± 0.01221	1049	0.99509
	Non-chemotherapy drugs	−0.00556 ± 0.02026	120	−0.00086 ± 0.01872	2760	0.03937
ATC classification	Immunostimulants	−0.02314 ± 0.03866	5	0.00916 ± 0.02849	115	0.00110
	Other dermatological preparations	−0.05846 ± ----------	1	−0.00353 ± 0.01580	23	0.01755
Transcription factor level	Directly	−0.00433 ± 0.02310	60	0.00070 ± 0.01671	1378	0.22309
	Not-directly	−0.00344 ± 0.01443	98	−0.00116 ± 0.01785	2224	0.99509
	Non-Human	−0.00533±0.01574	9	−0.00057 ± 0.01627	207	0.79080

Important subgroups or subgroups with false discover rate (FDR) q-value lower than 0.05 from GLM least squares mean partitions F tests for signed Jaccard index differences between drug-indicted disease pairs and random drug–disease pairs. “----------” indicates that subgroups only have one unique drug–disease pair sample with no standard deviation.

**Table 3:** Top 5% genes with relatively expression probability (*G^I-R%^*).

Gene	*G^I-R%^*	Gene	*G^I-R%^*	Gene	*G^I-R%^*	Gene	*G^I-R%^*
MX1	−46.87%	FTL	−25.22%	USP18	−19.56%	DUSP6	−16.90%
IFIT3	−41.45%	RPL24	−25.18%	CERS2	−19.38%	TPT1	−16.66%
NME1	−40.50%	ERP29	−23.86%	RPLP0	−19.36%	RSAD2	−16.59%
RPL3	−39.19%	RSL24D1	−23.86%	KLRB1	−19.28%	ADAR	−16.48%
RPS5	−37.61%	PTMA	−23.65%	ADM	−19.23%	DDX58	−16.44%
RPL6	−36.57%	HLA-DRA	−22.88%	PLSCR1	−19.23%	APOBEC3A	−16.40%
MT1HL1	−35.52%	IFIT1	−22.22%	RPLP0P6	−19.14%	PPIB	−16.17%
MT2A	−34.80%	MX2	−22.22%	RPS3A	−19.07%	RGS2	−16.09%
RPSA	−33.55%	LDHB	−22.12%	TRIM22	−19.00%	IRF7	−16.08%
TGFBI	−33.47%	DYNLT1	−21.90%	DDX21	−18.66%	PSMA6	−16.00%
MT1X	−32.30%	ALDH1A1	−21.64%	GCH1	−18.64%	RPL9	−15.94%
HERC5	−32.15%	HSPA1A	−21.53%	GAPDH	−18.55%	OAS1	−15.91%
FAU	−31.82%	SLC25A5	−21.53%	OAS3	−18.48%	RPL31	−15.74%
PLS3	−29.66%	IFIT2	−21.38%	RPS25	−18.40%	PTTG1IP	−15.74%
HLA-A	−29.15%	RPS4X	−21.28%	NDUFB11	−18.40%	BIRC2	−15.74%
RPL22	−28.88%	EIF3E	−20.88%	SNHG6	−18.15%	MYD88	−15.67%
FBL	−28.52%	HMGN2	−20.88%	PSAT1	−18.06%	RPS14P3	−15.64%
RPS8	−27.57%	FTH1P5	−20.80%	IER2	−18.02%	FTH1	−15.62%
ISG15	−26.91%	YWHAZ	−20.72%	UXT	−17.65%	C4orf46	−15.45%
EEF1B2	−26.88%	PFDN5	−20.57%	PARP12	−17.58%	PPT1	−15.42%
PHB2	−26.48%	TMA7	−20.20%	MAFB	−17.40%	YBX1	−15.33%
MT1H	−26.29%	CCT7	−20.12%	LYZ	−17.25%		
RPL8	−26.11%	OASL	−19.89%	NARS	−17.15%		
ATF4	−25.36%	SNHG5	−19.64%	AKR1B1	−17.02%		

**Table 4:** Top 10 significant biological pathways according to high relatively expression probability genes.

Ingenuity Canonical Pathways	-log(p-value)	Ratio	Genes Overlapped with Datasets
EIF2 Signaling	16.50	8.02% (17/212)	ATF4, EIF3E, FAU, RPL22, RPL24, RPL3, RPL31, RPL6, RPL8, RPL9, RPLP0, RPS25, RPS3A, RPS4X, RPS5, RPS8, RPSA
Activation of IRF by Cytosolic Pattern Recognition Receptors	6.60	9.84% (6/61)	ADAR, DDX58, IFIT2, IRF7, ISG15, PPIB
Regulation of eIF4 and p70S6K Signaling	6.48	5.23% (8/153)	EIF3E, FAU, RPS25, RPS3A, RPS4X, RPS5, RPS8, RPSA
Interferon Signaling	6.34	13.90% (5/36)	IFIT1, IFIT3, ISG15, MX1, OAS1
mTOR Signaling	5.57	3.96% (8/202)	EIF3E, FAU, RPS25, RPS3A, RPS4X, RPS5, RPS8, RPSA
NRF2-mediated Oxidative Stress Response	3.80	3.23% (6/186)	ATF4, CCT7, ERP29, FTH1, FTL, PPIB
Role of Pattern Recognition Receptors in Recognition of Bacteria and Viruses	3.39	3.47% (5/144)	DDX58, IRF7, MYD88, OAS1, OAS3
Neuroinflammation Signaling Pathway	2.78	2.06% (6/291)	ATF4, BIRC2, HLA-A, HLA-DRA, IRF7, MYD88
SPINK1 General Cancer Pathway	2.63	4.92% (3/61)	MT1H, MT1X, MT2A
Systemic Lupus Erythematosus in B Cell Signaling Pathway	2.23	1.89% (5/265)	IFIT2, IFIT3, IRF7, ISG15, MYD88

**Table 5:** Top 10 pathways and their function labels.

Ingenuity Canonical Pathways	Function	Reference
EIF2 Signaling	Immune Responses	[28]
Activation of IRF by Cytosolic Pattern Recognition Receptors	Regulate Interferon	[17]
Regulation of eIF4 and p70S6K Signaling	Inflammatory	[18,29]
Interferon Signaling	Immune Responses	[30,31]
mTOR Signaling	Immune Responses	[19]
NRF2-mediated Oxidative Stress Response	Antioxidant Response	[21]
Role of Pattern Recognition Receptors in Recognition of Bacteria and Viruses	Regulate Interferon	[22]
Neuroinflammation Signaling Pathway	Inflammatory	[23]
SPINK1 General Cancer Pathway	Cancer Diagnose	[32]
Systemic Lupus Erythematosus in B Cell Signaling Pathway	Inflammatory	[33]

## Supplementary Materials

The following are available online at https://www.mdpi.com/1420-3049/25/12/2776/s1, Table S1: 70 subgroups with four drug categories, Table S2: 70 subgroups with disease category, Table S3: Indicated drug–disease pair results, Table S4: Random drug–disease pair results.
