# Supplementary material for: The Performance of Gene Expression Signature-Guided Drug–Disease Association in Different Categories of Drugs and Diseases
Source: Molecules. 2020 Jun 16;25(12):2776. doi: 10.3390/molecules25122776 (PMC7357095; doi:10.3390/molecules25122776)
Supplement: Supplementary file 1 [file molecules-25-02776-s001.pdf]

The Performance of Gene Expression Signature-Guided Drug–Disease Association in Different Categories of Drugs and Diseases

Xiguang Qi [1], Mingzhe Shen [1], Peihao Fan [1], Xiaojiang Guo [1], Tianqi Wang [2], Ning Feng [3], Manling Zhang [3], Robert A. Sweet [4,5,*], Levent Kirisci [6,*] and Lirong Wang [1,*]

[1] Department of Pharmaceutical Sciences, Computational Chemical Genomics Screening, University of Pittsburgh School of Pharmacy, 3501 Terrace St Pittsburgh, PA 15261, USA
[2] Department of Biological Sciences, University of Pittsburgh School of Arts & Sciences, Pittsburgh, PA, 15260, USA
[3] Division of Cardiology, Vascular Medicine Institute, University of Pittsburgh School of Medicine, Pittsburgh, PA, 15213, USA
[4] Department of Neurology, University of Pittsburgh School of Medicine, Pittsburgh, PA, 15213, USA
[5] Department of Psychiatry, University of Pittsburgh School of Medicine, Pittsburgh, PA, 15213, USA
[6] Department of Pharmaceutical Sciences, University of Pittsburgh School of Pharmacy, 3501 Terrace St Pittsburgh, PA 15261, USA
* Correspondence: L.W., liw30@pitt.edu; Tel.: +1 412-624-8118 L.K., levent@pitt.edu; Tel.: +1 412-383-6089 R.A.S., sweetra@upmc.edu; Tel.: +1 412-624-0064

Table S1. 70 subgroups with four drug categories.

UNIFIED Drug Name	TF Level	Target	chemotherapy	ATC Classification
Abiraterone	not-directly	Cytochromes P450	NO	Hormone antagonists and related agents
Actinomycin d	not-directly	DNA/topoisomerase-human	YES	Cytotoxic antibiotics and related substances
Aminolevulinic acid	not-directly	Delta-aminolevulinic acid dehydratase	NO	Other antineoplastic agents
Anastrozole	not-directly	Cytochromes P450	NO	Hormone antagonists and related agents
Atorvastatin	not-directly	HMG-CoA reductase	NO	Lipid modifying agents, plain
Azacitidine	directly	DNA/methyltransferase	YES	Antimetabolites
Bexarotene	directly	Retinoic acid receptor	NO	Other antineoplastic agents
Bicalutamide	directly	Androgen receptor	NO	Hormone antagonists and related agents
Bleomycin	not-directly	DNA/ligase	YES	Cytotoxic antibiotics and related substances
Bortezomib	not-directly	Proteasome subunit beta	NO	Other antineoplastic agents
Calcitriol	directly	Vitamin D3 receptor	NO	Vitamin a and d, incl. combinations of the two

UNIFIED Drug Name	TF Level	Target	chemotherapy	ATC Classification
Carboplatin	not-directly	DNA cross-linking/alkylation	YES	Other antineoplastic agents
Celecoxib	not-directly	cyclooxygenase	NO	Anti-inflammatory and antirheumatic products, non-steroids
Chlorambucil	not-directly	DNA cross-linking/alkylation	YES	Alkylating agents
Ciprofloxacin	non-Human	topoisomerase-non-Human	NO	Quinolone antibacterials
Cisplatin	not-directly	DNA cross-linking/alkylation	YES	Other antineoplastic agents
Cytarabine	not-directly	DNA/polymerase	YES	Antimetabolites
Dasatinib	not-directly	tyrosine kinase	NO	Other antineoplastic agents
Decitabine	directly	DNA/methyltransferase	YES	Antimetabolites
Dexamethasone	directly	Glucocorticoid receptor	NO	Corticosteroids for systemic use, plain
Diclofenac	not-directly	cyclooxygenase	NO	Anti-inflammatory and antirheumatic products, non-steroids
Doxorubicin	not-directly	DNA/topoisomerase-human	YES	Cytotoxic antibiotics and related substances
Doxycycline	non-Human	16S ribosomal RNA	NO	Tetracyclines
Estradiol	directly	Estrogen receptor	NO	Estrogens
Etanercept	not-directly	Tumor necrosis factor	NO	Immunosuppressants
Fluorouracil	not-directly	Thymidylate synthase	YES	Antimetabolites
Formoterol	not-directly	Beta adrenergic receptor	NO	Adrenergics, inhalants
Gatifloxacin	non-Human	topoisomerase-non-Human	NO	Quinolone antibacterials
Gefitinib	not-directly	tyrosine kinase	NO	Other antineoplastic agents
Hydrocortisone	directly	Glucocorticoid receptor	NO	Corticosteroids for systemic use, plain
Hydroxyzine	not-directly	Histamine H1 receptor	NO	Anxiolytics
Imatinib	not-directly	tyrosine kinase	NO	Other antineoplastic agents
Insulin	not-directly	Insulin receptor	NO	Insulins and analogues

UNIFIED Drug Name	TF Level	Target	chemotherapy	ATC Classification
Interferon beta-1a	not-directly	Interferon receptor	NO	Immunostimulants
Interferon beta-1b	not-directly	Interferon receptor	NO	Immunostimulants
Interferon gamma-1b	not-directly	Interferon receptor	NO	Immunostimulants
Isotretinoin	directly	Retinoic acid receptor	NO	Anti-acne preparations for systemic use
Lapatinib	not-directly	tyrosine kinase	NO	Other antineoplastic agents
Letrozole	not-directly	Cytochromes P450	NO	Hormone antagonists and related agents
Levofloxacin	non-Human	topoisomerase-non-Human	NO	Quinolone antibacterials
Metformin	not-directly	AMP-activated protein kinase	NO	Blood glucose lowering drugs, excl. insulins
Methotrexate	not-directly	nucleotide synthesis	YES	Antimetabolites
Methotrexate	not-directly	aminoimidazole caboxamide ribonucleotide transformylase	NO	Immunosuppressants
Metoprolol	not-directly	Beta adrenergic receptor	NO	Beta blocking agents
Natural alpha interferon	not-directly	Interferon receptor	NO	Immunostimulants
Nilotinib	not-directly	tyrosine kinase	NO	Other antineoplastic agents
Paclitaxel	not-directly	microtubules	YES	Plant alkaloids and other natural products
Pertuzumab	not-directly	tyrosine kinase	NO	Other antineoplastic agents
Pimecrolimus	not-directly	kinase mTOR	NO	Other dermatological preparations
Pioglitazone	directly	Peroxisome proliferator-activated receptors	NO	Blood glucose lowering drugs, excl. insulins
Ribavirin	non-Human	Inosine-5'-monophosphate dehydrogenase	NO	Direct acting antivirals
Rituximab	not-directly	CD20 antigen	NO	Other antineoplastic agents
Rosiglitazone	directly	Peroxisome proliferator-activated receptors	NO	Blood glucose lowering drugs, excl. insulins
Sorafenib	not-directly	tyrosine kinase	NO	Other antineoplastic agents
Tamoxifen	directly	Estrogen receptor	NO	Hormone antagonists and related agents

UNIFIED Drug Name	TF Level	Target	chemotherapy	ATC Classification
Temozolomide	not-directly	DNA cross-linking/alkylation	YES	Alkylating agents
Trastuzumab	not-directly	tyrosine kinase	NO	Other antineoplastic agents
Tretinoin	directly	Retinoic acid receptor	NO	Anti-acne preparations for systemic use
Vemurafenib	not-directly	Serine/threonine-protein kinase B-raf	NO	Other antineoplastic agents

Table S2. 70 subgroups with disease category.

UNIFIED disease name	Classification
bacterial infectious disease	Certain infectious or parasitic diseases
hepatitis C	Certain infectious or parasitic diseases
Septic Shock	Certain infectious or parasitic diseases
aplastic anemia	Diseases of the blood or blood-forming organs
autoimmune thrombocytopenic purpura	Diseases of the blood or blood-forming organs
Diamond-Blackfan anemia	Diseases of the blood or blood-forming organs
acute myocardial infarction	Diseases of the circulatory system
ulcerative colitis	Diseases of the digestive system
childhood type dermatomyositis	Diseases of the immune system
chronic granulomatous disease	Diseases of the immune system
dermatomyositis	Diseases of the immune system
Polymyositis	Diseases of the immune system
pulmonary sarcoidosis	Diseases of the immune system
sarcoidosis	Diseases of the immune system
systemic lupus erythematosus	Diseases of the immune system
ankylosing spondylitis	Diseases of the musculoskeletal system or connective tissue
juvenile rheumatoid arthritis	Diseases of the musculoskeletal system or connective tissue
osteoarthritis	Diseases of the musculoskeletal system or connective tissue
osteoporosis	Diseases of the musculoskeletal system or connective tissue
psoriatic arthritis	Diseases of the musculoskeletal system or connective tissue
rheumatoid arthritis	Diseases of the musculoskeletal system or connective tissue
multiple sclerosis	Diseases of the nervous system
relapsing-remitting multiple sclerosis	Diseases of the nervous system
allergic asthma	Diseases of the respiratory system

asthma	Diseases of the respiratory system
chronic obstructive pulmonary disease	Diseases of the respiratory system
acne	Diseases of the skin
actinic keratosis	Diseases of the skin
allergic contact dermatitis	Diseases of the skin
atopic dermatitis	Diseases of the skin
discoid lupus erythematosus	Diseases of the skin
psoriasis	Diseases of the skin
urticaria	Diseases of the skin
familial hypercholesterolemia	Endocrine, nutritional or metabolic diseases
type 1 diabetes mellitus	Endocrine, nutritional or metabolic diseases
type 2 diabetes mellitus	Endocrine, nutritional or metabolic diseases
acute myeloid leukemia	Neoplasms
anaplastic thyroid carcinoma	Neoplasms
astrocytoma	Neoplasms
breast cancer	Neoplasms
chronic myeloid leukemia	Neoplasms
colon cancer	Neoplasms
ductal carcinoma in situ	Neoplasms
esophagus adenocarcinoma	Neoplasms
esophagus squamous cell carcinoma	Neoplasms
gastrointestinal stromal tumor	Neoplasms
glioblastoma multiforme	Neoplasms
head and neck squamous cell carcinoma	Neoplasms
hepatocellular carcinoma	Neoplasms
LGLL - Large granular lymphocytic leukemia	Neoplasms
lung adenocarcinoma	Neoplasms
lung large cell carcinoma	Neoplasms
lung small cell carcinoma	Neoplasms
lung squamous cell carcinoma	Neoplasms
melanoma	Neoplasms
multiple myeloma	Neoplasms
myelodysplastic syndrome	Neoplasms
nephroblastoma	Neoplasms
ovarian cancer	Neoplasms
ovarian serous carcinoma	Neoplasms
pancreatic cancer	Neoplasms
papillary thyroid carcinoma	Neoplasms
precursor B lymphoblastic lymphoma/leukemia	Neoplasms
prostate cancer	Neoplasms
renal cell carcinoma	Neoplasms

skin squamous cell carcinoma	Neoplasms
Squamous cell carcinoma of mouth	Neoplasms
testicular cancer	Neoplasms
testis seminoma	Neoplasms
chronic lymphocytic leukemia	Neoplasms
Leukemia, Chronic T-Cell	Neoplasms

Table S3. Indicated drug–disease pair results.

Category	Subgroups	Size	SD	Mean
Target	16S ribosomal RNA	2	0.00144	0.00134
Target	aminoimidazole caboxamide ribonucleotide transformylase	4	0.00656	-0.00218
Target	AMP-activated protein kinase	1	----	0.00198
Target	Androgen receptor	1	----	0.01733
Target	Beta adrenergic receptor	4	0.01611	0.00012
Target	CD20 antigen	3	0.00233	-0.00057
Target	cyclooxygenase	8	0.01331	-0.00293
Target	Cytochromes P450	3	0.01320	-0.00253
Target	Delta-aminolevulinic acid dehydratase	1	----	0.00974
Target	Estrogen receptor	4	0.00589	0.00107
Target	Glucocorticoid receptor	48	0.02451	-0.00715
Target	HMG-CoA reductase	1	----	-0.00594
Target	Inosine-5'-monophosphate dehydrogenase	1	----	-0.04285
Target	Insulin receptor	2	0.00157	-0.00259
Target	Interferon receptor	5	0.03866	-0.02314
Target	kinase mTOR	1	----	-0.05846
Target	Peroxisome proliferator-activated receptors	1	----	0.00390
Target	Proteasome subunit beta	1	----	0.00923
Target	Retinoic acid receptor	3	0.01861	0.01548
Target	Serine/threonine-protein kinase B-raf	1	----	-0.00551
Target	topoisomerase-non-Human	6	0.00879	-0.00129
Target	Tumor necrosis factor	5	0.00965	-0.00473
Target	tyrosine kinase	13	0.01001	-0.00917
Target	Vitamin D3 receptor	1	----	0.00537
Target	DNA cross-linking/alkylation	10	0.00989	-0.00067
Target	DNA/ligase	6	0.00368	0.00084
Target	DNA/methyltransferase	2	0.00561	0.00305
Target	DNA/polymerase	2	0.01131	-0.01287
Target	DNA/topoisomerase-human	13	0.00915	-0.00053
Target	microtubules	5	0.00665	0.01363
Target	Thymidylate synthase	4	0.00182	-0.00033

Target	nucleotide synthesis	5	0.00334	-0.00323
ATC Classification	tetracyclines	2	0.00144	0.00134
ATC Classification	immunosuppressants	9	0.00803	-0.00360
ATC Classification	blood glucose lowering drugs, excl. insulins	2	0.00136	0.00294
ATC Classification	hormone antagonists and related agents	6	0.01166	0.00343
ATC Classification	adrenergics, inhalants	3	0.00292	0.00816
ATC Classification	beta blocking agents	1	----	-0.02369
ATC Classification	other antineoplastic agents	26	0.01008	-0.00467
ATC Classification	Anti-inflammatory and antirheumatic products, non-steroids	8	0.01331	-0.00293
ATC Classification	estrogens	2	0.00221	-0.00327
ATC Classification	corticosteroids for systemic use, plain	48	0.02451	-0.00715
ATC Classification	lipid modifying agents, plain	1	----	-0.00594
ATC Classification	direct acting antivirals	1	----	-0.04285
ATC Classification	insulins and analogues	2	0.00157	-0.00259
ATC Classification	immunostimulants	5	0.03866	-0.02314
ATC Classification	other dermatological preparations	1	----	-0.05846
ATC Classification	anti-acne preparations for systemic use	2	0.02613	0.01675
ATC Classification	quinolone antibacterials	6	0.00879	-0.00129
ATC Classification	vitamin a and d, incl. combinations of the two	1	----	0.00537
ATC Classification	alkylating agents	4	0.01093	0.00502
ATC Classification	cytotoxic antibiotics and related substances	19	0.00775	-0.00010
ATC Classification	antimetabolites	13	0.00652	-0.00286
ATC Classification	plant alkaloids and other natural products	5	0.00665	0.01363
TF Level	non-Human	9	0.01574	-0.00533
TF Level	not-directly	98	0.01443	-0.00344
TF Level	directly	60	0.02310	-0.00433
Classification	Certain infectious or parasitic diseases	10	0.01500	-0.00600
Classification	Diseases of the blood or blood-forming organs	6	0.03746	-0.02368
Classification	Diseases of the circulatory system	1	----	-0.02369
Classification	Diseases of the digestive system	2	0.00078	-0.00297
Classification	Diseases of the immune system	10	0.01758	-0.00723
Classification	Diseases of the musculoskeletal system or connective tissue	26	0.00768	-0.00170
Classification	Diseases of the nervous system	4	0.03648	-0.03264
Classification	Diseases of the respiratory system	5	0.00792	0.00300
Classification	Diseases of the skin	16	0.02951	-0.00569
Classification	Endocrine, nutritional or metabolic diseases	5	0.00403	-0.00105
Classification	Neoplasms	82	0.01394	-0.00103
chemotherapy	NO	120	0.02026	-0.00556
chemotherapy	YES	47	0.00894	0.00048

Table S4. Random drug–disease pair results.

Category	Subgroups	Size	SD	Mean
Target	16S ribosomal RNA	46	0.00587	0.00106
Target	aminoimidazole caboxamide ribonucleotide transformylase	92	0.00741	-0.00223
Target	AMP-activated protein kinase	23	0.01326	-0.00637
Target	Androgen receptor	23	0.02626	-0.00085
Target	Beta adrenergic receptor	92	0.02759	-0.00149
Target	CD20 antigen	69	0.01436	0.00118
Target	cyclooxygenase	184	0.00887	-0.00064
Target	Cytochromes P450	69	0.02139	-0.00440
Target	Delta-aminolevulinic acid dehydratase	23	0.00706	-0.00008
Target	Estrogen receptor	92	0.01238	0.00637
Target	Glucocorticoid receptor	1104	0.01943	-0.00194
Target	HMG-CoA reductase	23	0.02600	-0.00562
Target	Inosine-5'-monophosphate dehydrogenase	23	0.01932	-0.00028
Target	Insulin receptor	46	0.01103	0.00196
Target	Interferon receptor	115	0.02849	0.00916
Target	kinase mTOR	23	0.01580	-0.00353
Target	Peroxisome proliferator-activated receptors	23	0.01289	0.00118
Target	Proteasome subunit beta	23	0.01031	-0.00219
Target	Retinoic acid receptor	69	0.01689	0.00131
Target	Serine/threonine-protein kinase B-raf	23	0.00903	-0.00396
Target	topoisomerase-non-Human	138	0.01749	-0.00121
Target	Tumor necrosis factor	115	0.01891	-0.00753
Target	tyrosine kinase	299	0.01544	-0.00471
Target	Vitamin D3 receptor	23	0.01184	-0.00267
Target	DNA cross-linking/alkylation	230	0.01040	0.00047
Target	DNA/ligase	137	0.00165	-0.00056
Target	DNA/methyltransferase	44	0.01455	-0.00180
Target	DNA/polymerase	44	0.01139	0.00073
Target	DNA/topoisomerase-human	286	0.01900	-0.00090
Target	microtubules	110	0.01101	0.00258
Target	Thymidylate synthase	88	0.00329	-0.00152
Target	nucleotide synthesis	110	0.00595	-0.00096
ATC Classification	tetracyclines	46	0.00587	0.00106
ATC Classification	immunosuppressants	207	0.01623	-0.00580
ATC Classification	blood glucose lowering drugs, excl. insulins	46	0.01357	-0.00260
ATC Classification	hormone antagonists and related agents	138	0.02098	-0.00202
ATC Classification	adrenergics, inhalants	69	0.02579	-0.00083
ATC Classification	beta blocking agents	23	0.02941	-0.00213

ATC Classification	other antineoplastic agents	598	0.01379	-0.00298
ATC Classification	Anti-inflammatory and antirheumatic products, non-steroids	184	0.00887	-0.00064
ATC Classification	estrogens	46	0.01443	0.00869
ATC Classification	corticosteroids for systemic use, plain	1104	0.01943	-0.00194
ATC Classification	lipid modifying agents, plain	23	0.02600	-0.00562
ATC Classification	direct acting antivirals	23	0.01932	-0.00028
ATC Classification	insulins and analogues	46	0.01103	0.00196
ATC Classification	immunostimulants	115	0.02849	0.00916
ATC Classification	other dermatological preparations	23	0.01580	-0.00353
ATC Classification	anti-acne preparations for systemic use	46	0.01748	0.00257
ATC Classification	quinolone antibacterials	138	0.01749	-0.00121
ATC Classification	vitamin a and d, incl. combinations of the two	23	0.01184	-0.00267
ATC Classification	alkylating agents	92	0.01068	0.00120
ATC Classification	cytotoxic antibiotics and related substances	423	0.01550	-0.00079
ATC Classification	antimetabolites	286	0.01150	-0.00108
ATC Classification	plant alkaloids and other natural products	110	0.01101	0.00258
TF Level	non-Human	207	0.01627	-0.00057
TF Level	not-directly	2224	0.01785	-0.00116
TF Level	directly	1378	0.01671	0.00070
Classification	Certain infectious or parasitic diseases	230	0.00999	0.00031
Classification	Diseases of the blood or blood-forming organs	138	0.02470	0.00075
Classification	Diseases of the circulatory system	23	0.01064	-0.00186
Classification	Diseases of the digestive system	46	0.00555	-0.00001
Classification	Diseases of the immune system	230	0.02018	0.00376
Classification	Diseases of the musculoskeletal system or connective tissue	598	0.01129	-0.00199
Classification	Diseases of the nervous system	92	0.01528	-0.00054
Classification	Diseases of the respiratory system	115	0.01108	-0.00278
Classification	Diseases of the skin	367	0.02185	-0.00204
Classification	Endocrine, nutritional or metabolic diseases	115	0.00930	-0.00084
Classification	Neoplasms	1855	0.01789	-0.00117
chemotherapy	NO	2760	0.01872	-0.00086
chemotherapy	YES	1049	0.01221	-0.00022

GLM least squares mean partitions F tests results.

Category	Subgroups	p-value	FDR
ATC Classification	adrenergics, inhalants	0.374715709	0.995085228
ATC Classification	alkylating agents	0.660522074	0.995085228
ATC Classification	anti-acne preparations for systemic use	0.246044488	0.854417604

ATC Classification	Anti-inflammatory and antirheumatic products, non-steroids	0.713943031	0.995085228
ATC Classification	antimetabolites	0.715762413	0.995085228
ATC Classification	beta blocking agents	0.212485563	0.790803013
ATC Classification	blood glucose lowering drugs, excl. insulins	0.650599990	0.995085228
ATC Classification	corticosteroids for systemic use, plain	0.087245247	0.469782099
ATC Classification	cytotoxic antibiotics and related substances	0.866263846	0.995085228
ATC Classification	direct acting antivirals	0.013876595	0.107929072
ATC Classification	estrogens	0.331382166	0.966531318
ATC Classification	hormone antagonists and related agents	0.441362019	0.995085228
ATC Classification	immunostimulants	0.000031353	0.001097355
ATC Classification	immunosuppressants	0.712747738	0.995085228
ATC Classification	insulins and analogues	0.712189994	0.995085228
ATC Classification	lipid modifying agents, plain	0.985052852	0.995085228
ATC Classification	other antineoplastic agents	0.620012925	0.995085228
ATC Classification	other dermatological preparations	0.001503920	0.017545733
ATC Classification	plant alkaloids and other natural products	0.165302998	0.723200616
ATC Classification	quinolone antibacterials	0.991273187	0.995085228
ATC Classification	tetracyclines	0.981997017	0.995085228
ATC Classification	vitamin a and d, incl. combinations of the two	0.642022253	0.995085228
chemotherapy	NO	0.003936611	0.039366110
chemotherapy	YES	0.789735814	0.995085228
Disease Classification	Certain infectious or parasitic diseases	0.266804359	0.854417604
Disease Classification	Diseases of the blood or blood-forming organs	0.000755394	0.013219395
Disease Classification	Diseases of the circulatory system	0.214646532	0.790803013
Disease Classification	Diseases of the digestive system	0.813739044	0.995085228
Disease Classification	Diseases of the immune system	0.049131706	0.312656311
Disease Classification	Diseases of the musculoskeletal system or connective tissue	0.936781579	0.995085228
Disease Classification	Diseases of the nervous system	0.000301680	0.007039200
Disease Classification	Diseases of the respiratory system	0.464962229	0.995085228
Disease Classification	Diseases of the skin	0.411044369	0.995085228
Disease Classification	Endocrine, nutritional or metabolic diseases	0.979119265	0.995085228
Disease Classification	Neoplasms	0.942110224	0.995085228

Target	16S ribosomal RNA	0.981961009	0.995085228
Target	AMP-activated protein kinase	0.628327905	0.995085228
Target	Androgen receptor	0.292209117	0.889332095
Target	Beta adrenergic receptor	0.845985358	0.995085228
Target	CD20 antigen	0.862365043	0.995085228
Target	Cytochromes P450	0.851301078	0.995085228
Target	DNA cross-linking/alkylation	0.835585627	0.995085228
Target	DNA/ligase	0.847800445	0.995085228
Target	DNA/methyltransferase	0.691339243	0.995085228
Target	DNA/polymerase	0.268531247	0.854417604
Target	DNA/topoisomerase-human	0.939599173	0.995085228
Target	Delta-aminolevulinic acid dehydratase	0.569461927	0.995085228
Target	Estrogen receptor	0.541953892	0.995085228
Target	Glucocorticoid receptor	0.086615134	0.469782099
Target	HMG-CoA reductase	0.985022954	0.995085228
Target	Inosine-5'-monophosphate dehydrogenase	0.013687736	0.107929072
Target	Insulin receptor	0.711639979	0.995085228
Target	Interferon receptor	0.000030234	0.001097355
Target	Peroxisome proliferator-activated receptors	0.874662720	0.995085228
Target	Proteasome subunit beta	0.508377757	0.995085228
Target	Retinoic acid receptor	0.155179037	0.723200616
Target	Serine/threonine-protein kinase B-raf	0.928537948	0.995085228
Target	Thymidylate synthase	0.892310400	0.995085228
Target	Tumor necrosis factor	0.725131277	0.995085228
Target	Vitamin D3 receptor	0.641356423	0.995085228
Target	aminoimidazole caboxamide ribonucleotide transformylase	0.995085228	0.995085228
Target	cyclooxygenase	0.713396047	0.995085228
Target	kinase mTOR	0.001471446	0.017545733
Target	microtubules	0.164459273	0.723200616
Target	nucleotide synthesis	0.783282308	0.995085228
Target	topoisomerase-non-Human	0.991255730	0.995085228
Target	tyrosine kinase	0.354435901	0.992420523
TF-level	directly	0.031870637	0.223094459
TF-level	non-Human	0.421462012	0.995085228
TF-level	not-directly	0.205022051	0.790803013
